# Unmet Needs for Aging Care Assistance Among Community-Dwelling Older Adults: A Life Course Investigation

**DOI:** 10.1093/geroni/igaf122.4227

**Published:** 2025-12-31

**Authors:** Millicent Ngwu

**Affiliations:** Department of Sociology and Criminology University at Buffalo, Buffalo, New York, United States

## Abstract

Unmet care needs are a growing public health and policy issue across developed countries. Older adults are faced with the challenge of maintaining quality care. The unmet need for aging care assistance is shaped by social identities, including gender and race. Although early life disadvantages shape later life outcomes, little is known about how childhood experiences matter for unmet care needs among community-dwelling older adults. Guided by the life course perspective, this study examined how early life experiences, such as childhood financial background and health status, matter for unmet care needs. The data for this study were drawn from Round 12 of the National Health and Aging Trends Study (2022) to statistically analyze how early life experiences matter for unmet care needs among community-dwelling older adults. The study’s analysis revealed that early life experiences, such as fair or poor childhood health status, remained a significant predictor of unmet care needs in later life, with fair or poor health status significantly linked to increased odds of older adults having unmet needs for aging care assistance. Sociodemographic factors such as age, education, adulthood health status and income significantly predict unmet needs. Further, as older adults advance in age, they continue to face increased odds of experiencing unmet needs. The policy implications include prioritizing health programs through targeted policies for children and adults who have poor health status.

